# Safety and tolerability of intramuscular and sublingual ketamine for psychiatric treatment in the Roots To Thrive ketamine-assisted therapy program: a retrospective chart review

**DOI:** 10.1177/20451253231171512

**Published:** 2023-05-25

**Authors:** Vivian W.L. Tsang, Brendan Tao, Shannon Dames, Zach Walsh, Pam Kryskow

**Affiliations:** Department of Psychiatry, The University of British Columbia, 2255 Wesbrook Mall, Vancouver, BC V6T 2A1, Canada; Faculty of Medicine, The University of British Columbia, Vancouver, BC, Canada; Health Sciences and Human Services, Vancouver Island University, Nanaimo, BC, Canada; Department of Psychology, The University of British Columbia, Kelowna, BC, Canada; Department of Family Medicine, The University of British Columbia, Vancouver, BC, Canada

**Keywords:** depression, group therapy, ketamine, psychedelic, psychotherapy

## Abstract

**Background:**

In the last few years, ketamine is becoming increasingly common in the treatment of mental health conditions, but there is a lack of safety data informing intramuscular and sublingual dosing in a community-focused group psychotherapy setting. The Roots To Thrive ketamine-assisted therapy (RTT-KaT) program is a unique 12-week RTT-KaT program with 12 community of practice (a form of group therapy) sessions and three ketamine medicine sessions.

**Objectives:**

This study reports on adverse effects of intramuscular and sublingual ketamine dosing in a community group psychotherapy setting among 128 participants across four cohorts.

**Design:**

Retrospective chart review.

**Methods:**

A chart review of the RTT-KaT Program was performed retrospectively on four cohorts (*n* = 128) that participated in 448 sessions running between September 2020 and December 2021. Baseline characteristics and adverse events were captured including medication administration before, during, and after RTT-KaT sessions. Analyses by session and by individual were conducted. Chi-square test with Yates’ continuity correction was used to assess side effects in subgroups from ketamine administration.

**Results:**

RTT-KaT was well tolerated with none of the 128 participants dropping out of the program. Primarily, of the 448 sessions, 49.16% had elevated blood pressures post-KaT session by session. In terms of other adverse effects, 12.05% of participant-sessions experienced nausea, 2.52% had an episode of vomiting, 3.35% had a headache, and seven participant-sessions experienced dizziness. Analysis by individual revealed congruent findings.

**Conclusion:**

These findings suggest good safety and tolerability for RTT-KaT among individuals seeking treatment for mental health issues. The majority of participants did not experience adverse reactions and the adverse events that were recorded involved transient symptoms that were resolved with rest and/or medications. The group therapy model described provides a comprehensive approach and presents a promising model for operating a KaT program in a community setting.

## Introduction

Although ketamine has been used safely as an analgesic and anesthetic in operating rooms for decades, its use in treating mental health conditions is relatively new.^1^ Ketamine-assisted therapy (KaT) for psychiatric disorders has been used since the 1970s for treating depression and alcohol use disorder.^2–6^ More recently, the use of intravenous ketamine for chronic pain or depression has also gained popularity. Generic ketamine has been used for decades in KaT in sublingual and intramuscular form in the community by skilled therapists with good results.^7^ KaT has been proposed to work by re-orienting participants away from unhelpful patterns toward empowered awareness and more secure ways of being.^8^

Currently, ketamine’s drug monograph lists the following as incidents of adverse drug reactions in >10%: hypertension, increased cardiac output, increased intracranial pressure, tachycardia, tonic-clonic movements, visual hallucinations, vivid dreams; in 1–10%: bradycardia, diplopia, hypotension, increased intraocular pressure, injection site pain, nystagmus; and in < 1%: anaphylaxis, cardiac arrhythmia, cough reflex depressed, fasciculations, hypersalivation, increased metabolic rate, hypertonia, laryngospasm, respiratory depression, apnea.^9^

There is ample safety data published on the use of both intravenous and intranasal ketamine for treatment-resistant depression.^10–18^ A formulation of intranasal ketamine received an on-label indication for use in treatment of depression and suicidal ideation in 2020.^19^ Along with this approval comes documentation from funded trials sponsored by a pharmaceutical company.^20^ Although secondary reviews exist, the generic status of racemic ketamine may reduce incentives for costly clinical trials to establish the safety and efficacy of intramuscular and sublingual administration for psychiatric disorders.^21^

The Roots To Thrive KaT (RTT-KaT) program has been operating since 2019 and has documented the clinical ketamine sessions of over 212 patients. Patients who qualify for the program are diagnosed with at least one of the following conditions: post-traumatic stress disorder (PTSD), depression, generalized anxiety, burnout/adjustment disorder, substance use disorder (SUD), obsessive compulsive disorder (OCD), disordered eating, and disordered sleep. A detailed account of the program has been detailed elsewhere.^8^

The rigor and structure of the RTT-KaT program coupled with data spanning over 2 years ensures reliable and accessible analyses. Based on data from 128 participants, this article identifies and analyzes the adverse effects to provide preliminary safety data to inform subsequent researchers and providers using KaT to treat psychiatric disorders. To date, while ample safety data exist for ketamine and its various routes of administration, there is little related evidence for its safety in a novel community group psychotherapy setting, referred to hereafter as a community of practice (CoP). Therefore, the rationale for this study is to evaluate the safety and tolerability of sublingual and intramuscular ketamine in a community using the novel RTT-KaT program.

## Methods

### Participants

A retrospective chart review of ketamine session notes was conducted from cohorts 1–4 of the RTT-KaT program. The four cohorts ran from September 2020 to December 2021 and included 128 participants. Cohorts one through four had 17, 39, 52, and 49 unique participants, respectively, with some subjects as unique participants in multiple cohorts. Written informed consent was obtained from all participants upon voluntary participation in the RTT-KaT program.

### Preparation and setting

Participants in the RTT-KaT are referred by their primary health care provider or mental health provider. Three screening intakes with a patient navigator, medical practitioner, and psychiatrist are conducted before a participant is enrolled into the program. Exclusion criteria include uncontrolled or untreated hypertension (i.e. above 150/90), hepatic disorders, kidney disorders, some vascular disorders (reviewed case by case), presence of active psychotic symptoms, dementia, delirium, inability to tolerate group work, extreme emotional instability, ketamine allergy, and pregnancy. Medications known to interact with ketamine such as benzodiazepines and lamotrigine are weaned off (if possible) under the supervision of the participant’s psychiatrist or primary healthcare provider.^22^

Prior to any therapy or medicine session, participants receive a full presentation on KaT with opportunities to submit anonymous questions and participate in an online question and answer session. In addition, all participants have the opportunity for at least two private sessions with the program’s medical doctor to ensure all their questions are answered; that they understand the consent for treatment, know what to expect; and how they will be supported prior, during, and after their session. This is also reinforced in small group work that is done weekly.

The current RTT-KaT is a unique 12-week program with 12 CoP group therapy sessions and three ketamine medicine sessions. The CoP sessions provide support and resilience development among participants, focused on expanding conscious awareness, nervous system regulation, self and other compassion, and alignment with one’s calling (purpose/meaning). This research-informed group model addresses trauma and relational attachment issues and provides a practice environment that promotes the integration of learning into daily life.

### Ketamine sessions

In cohorts 1, 2, and 3, the first ketamine session was administered via sublingual formulation in a lozenge with 100–300 mg of ketamine. The lozenges, at the time of administration, were taken orally in the mouth, broken up, and swished around for 20 min and then spit out. Cohorts 1, 2, and 3 engaged in their second and third ketamine sessions via an intramuscular, gluteal, or deltoid injection of ketamine in the weight-based range of 0.5–1.5 mg/kg. Doses ranged from 45 to 145 mg. Shared decision-making between the supervising physician and patient occurred to modify dosages as needed in accordance with patient comfort and intentions for their session. Considerations include patient demographics, past experience with ketamine and past dosage used, and the presence of underlying health conditions.

For cohort 4, all three ketamine sessions were delivered intramuscularly due to participant feedback from previous cohorts, enhanced dosing precision, bioavailability, and feasibility of administration.

As standard RTT procedure, in all sessions where ketamine is administered, blood pressure and heart rate are taken prior to medicine administration. Should a patient arrive with blood pressures above the allowed cut-off (150/90), they will be brought to a private room to sit quietly for 5 min doing relaxation exercises. If a repeat blood pressure is still above cut-off, 0.1–0.2 mg of oral clonidine is provided and the patient is retested 20–30 min later. If the blood pressure is still above the cut-off, the participant is discharged home and ketamine is not administered.

After completing post-KaT initial integration, participants are picked up or have travel accommodations arranged for their safety. An emergency contact list is provided to allow all participants to reach three study team members at any time of day to discuss concerns related to the study. Participants are given the option of attending two or three more integration sessions prior to their next online RTT session.

Participants are coached on navigating challenging experiences under the influence of ketamine and are supported to integrate any challenging situation in follow-up integration sessions with the group with additional support as needed by team therapists, psychologists, and psychiatrists.

Before each session, patients and the care team are given the option to receive prophylactic medications for common adverse events of ketamine, including ondansetron, acetaminophen, ibuprofen, lorazepam, and clonidine. Given the observational nature of this study, the use of prophylactic medications is helpful to understand the side-effect profile of ketamine in this unique and real-world care setting, where prophylaxis is a common patient-oriented practice.

### Measures

Baseline demographics, adverse events, and pharmacotherapy required were abstracted from forms administered during ketamine sessions and inputted to the spreadsheet. Adverse events were assessed by the nurse staff and recorded during KaT and directly after treatment as well as 30 min post-KaT. All adverse effects, including expected and unexpected, were recorded as entries on these session forms, which were completed on the day of each ketamine administration.

Blood pressures were recorded before and after ketamine administration. First measurements were taken within 1 h prior to each ketamine session. Blood pressure was then retaken 20 min and again 2–3 h after administration, before participants left at the end of their session. Mental state was also collected on arrival and departure. Due to heterogeneity in the data entry, similar traits were categorized into either calm *versus* anxious *versus* N/A. Where items from more than one of these categories were entered, the first one reported was used.

### Data analysis

This study aimed to investigate the expected real-world adverse effects of KaT. Therefore, only descriptive statistics were used. In addition to reporting baseline characteristics of included participants, the overall incidence of adverse effects and the incidence stratified by baseline variables such as gender, age, and route of administration were reported.

Where applicable, baseline and outcome measures were analyzed by session (*n* = 448). Analysis and methods for analysis by individual is included in the Supplemental materials. In this article, unless otherwise specified, we report data by session as data were originally collected in this format and, therefore, permit greater resolution of study results. For transparency and brevity, we also report by-individual results in the Supplemental materials. After a review of the results, we determined that by-individual and by-session analyses yielded largely congruent safety findings.

Where applicable, the Chi-square test with Yates’ continuity correction was used to assess statistical significance for side effects in subgroups from ketamine administration. Analyses were performed in RStudio 2022.02.0+443.

## Results

### Study characteristics

RTT-KaT was well tolerated with no participants dropping out of the program. A total 128 participants (98 females; 30 males) contributed to 448 total sessions. Cohort 1 (*n* = 17) participated in 54 KaT sessions, cohort 2 (*n* = 39) in 128 sessions, cohort 3 (*n* = 52) in 143 sessions, and cohort 4 (*n* = 49) in 123 sessions. There were 29 instances of individuals participating in two cohorts. The average participant age and weight were 46.78 (SD 11.29) years and 74.28 (SD 15.45) kg, respectively. Tables 1 and 2 depict an overview of session characteristics stratified by age and gender. Participants reported feeling calm on arrival to 291 sessions and anxious to 77 sessions. For 80 participant-sessions, mood was unreported.

**Table 1.** Overall baseline features stratified by age cohort (by session).

Gender	20–30 years (*n* = 40 sessions)	31–40 years (*n* = 83)	41–50 years (*n* = 194)	51–60 years (*n* = 69)	61–70 years (*n* = 57)	71–80 years (*n* = 5)
	6 male sessions 34 female sessions	13 male sessions 70 female sessions	44 male sessions 150 female sessions	26 male sessions 43 female sessions	18 male sessions 39 female sessions	5 female sessions 0 male sessions
Age [mean (SD)]	27.65 (2.39)	36.40 (3.08)	44. 73 (2.87)	55.35 (2.75)	65. 96 (2.41)	73.00 (2.74)
Weight [mean (SD)]	78.31 (15.36)	69.17 (16.91)	74.26 (16.19)	76.26 (12.47)	78.10 (23.65)	86.06 (15.99)
Pre-BP [mean (SD)]	122.35/82.13 (12.57/12.25)	127.80/81.46 (15.36/10.16)	126.70/84.38 (15.75/12.22)	129.35/84.43 (12.23/11.34)	133.23/84.67 (16.92/10.31)	135/81.8 (4.82/7.71)
Pre-HTN prevalence (%)	20	32.93	38.02	39.13	47.37	40.00

BP, blood pressure; HTN, hypertension; SD, standard deviation.

Percentage figures exclude missing data.

**Table 2.** Overall baseline features stratified by gender (by session).

	Male (*N* = 107 sessions)	Female (*N* = 341 sessions)
Age [mean (SD)]	48.98 (11.18)	45.48 (11.25)
Weight [mean (SD)]	84.46 (12.15)	71.72 (17.41)
Pre-BP [mean (SD)]	134.83/86.93 (13.65/10.47)	125.64/82.62(15.02/11.57)
Pre-HTN (%)	54.21	31.36
IM *versus* SL	83 IM sessions 24 SL sessions	269 IM sessions 72 SL sessions

BP, blood pressure; HTN, hypertension; IM, intramuscular; SD, standard deviation; SL, sublingual.

There were 351 and 96 sessions using intramuscular ketamine or sublingual ketamine lozenges, respectively. One session was unreported. For the 351 intramuscular sessions, there were 31 male participants and 97 female participants. The average ketamine dose used in intramuscular sessions was 102.55 mg (SD 20.62 mg). The average pre-session blood pressure for these sessions was 128/84 (SD 16/11) and 37.71% of participant-sessions had elevated blood pressures prior to the KaT session. From a total of 96 sublingual sessions, there were 16 male and 53 female participants. The average ketamine dose used in sublingual sessions was 276.67 mg (SD 59.69 mg). The pre-session blood pressure for these sessions was 126/84 (SD 14/12) and 32.98% of participant-sessions were hypertensive prior to the KaT session.

Table 3 depicts the incidence of adverse events among included sessions. Accounting for missing data, 12.05% of 365 sessions reported nausea, 2.52% of 357 sessions had an episode of vomiting, 3.35% of 357 sessions reported headache, and. 1.56% of 448 sessions experienced dizziness. Also, among sessions that used medication prophylaxis, only a minority of these sessions had documented adverse events (38 of 105 sessions for nausea; 5 of 105 sessions for vomiting; 1 of 3 sessions for anxiousness; and zero of 13 sessions for post-treatment hypertension). However, headache prophylaxis was less effective as 10 of 14 sessions using acetaminophen and 4 of 13 sessions using ibuprofen still had this adverse event.

**Table 3.** Incidence of adverse events, use of medications in sessions with adverse events, and use of medications in all sessions.

Adverse event	Events (*n* = 448 sessions)	Use of drugs in sessions with AEs	Use of drugs in all sessions
Nausea	44 events, 321 event-free, 83 unreported	38/44 events used ondansetron (mean dose: 8 mg)	Ondansetron used in 105 sessions for nausea or another reason 12 mg (*n* = 4) 12.8 mg (*n* = 1) 16 mg (*n* = 3) 4 mg (*n* = 50) 8 mg (*n* = 47)
Vomiting	9 events, 348 event-free, 91 unreported	5/9 events used ondansetron (mean dose: 7.2 mg)	Ondansetron used in 105 sessions for vomiting or another reason 12 mg (*n* = 4) 12.8 mg (*n* = 1) 16 mg (*n* = 3) 4 mg (*n* = 50) 8 mg (*n* = 47)
Headache	12 events, 345 event-free, 91 unreported	10/12 events used acetaminophen (mean dose: 613.75 mg) 4 uses of ibuprofen (mean dose: 350 mg)	Acetaminophen used in 14 sessions for headache or another reason 1000 mg (*n* = 3) 487.5 mg (*n* = 1) 500 mg (*n* = 9) 650 mg (*n* = 1) Ibuprofen used in 13 sessions 200 mg (*n* = 3) 300 mg (*n* = 1) 400 mg (*n* = 9)
Anxiousness at departure	25 anxious, 363 calm, 60 unreported Calm = 363 (from 289 at baseline; 25.61% increase) Anxious = 25 (from 73 at baseline; 65.75% decrease) Unreported = 60 (from 86 at baseline; 30.23% decrease)	1/25 events used lorazepam (1 mg)	Used in three sessions for anxiousness on departure or another reason 0.5 mg (*n* = 2) 1 mg (*n* = 1)
Post-treatment HTN	204 events, 211 event-free, 33 unreported	No post-treatment therapy for HTN was used	Used in 13 sessions for post-treatment HTN or another reason 0.1 mg (*n* = 2) 0.2 mg (*n* = 11)

AE, adverse event; HTN, hypertension.

### Stratification by baseline differences

Comparing routes of administration, 13.26% of intramuscular sessions experienced nausea compared with 8.24% of sublingual sessions. Administration route was not significantly associated with incidence of nausea (χ^2^ = 1.11, *p* = 0.29). Intramuscular sessions had a higher incidence of headache (4.04% *versus* 1.19%) and dizziness (1.99% *versus* 0%) than sublingual sessions. However, sublingual sessions had higher rates of vomiting (1.85% *versus* 4.65%).

Table 4 summarizes the adverse effect measures by gender. Participant gender was significantly associated with incidence of nausea (χ^2^ = 4.28, *p* = 0.038), with lower incidence in males. Also, rates of nausea, vomiting, headache, and dizziness were similar across age cohorts (Table 5).

**Table 4.** Adverse effect measures stratified by gender (by session).

	Male (*n* = 107 sessions)	Female (*n* = 341 sessions)
Nausea (%)	5.43	13.89
Vomiting (%)	1.10	3.01
Headache (%)	0	4.51
Dizziness (%)	0	2.05
Post-elevated blood pressure	60.61% (Baseline: 54.21%)	45.60% (Baseline: 31.36%)
Post-session blood pressure [mean (SD)]	136/90 (21/17)	128/86 (18/14)

SD, standard deviation.

**Table 5.** Post-treatment measures by age (by session).

	20–30 years (*n* = 40 sessions)	31–40 years (*n* = 83 sessions)	41–50 years (*n* = 194 sessions)	51–60 years (*n* = 69 sessions)	61–70 years (*n* = 57 sessions)	71–80 years (*n* = 5 sessions)
Post-treatment elevated blood pressure	42.11% (Baseline: 20%)	41.56% (Baseline: 32.93%)	43.43% (Baseline: 38.02%)	60.29% (Baseline: 39.13%)	71.15% (Baseline: 47.37%)	40% (Baseline: 40%)
Post-session blood pressure [mean (SD)]	118/79 (16/13)	125/84 (19/13)	130/89 (17/15)	135/87 (21/16)	139/88 (21/14)	132/83 (21/12)
Nausea (%)	9.38	15.07	12.16	10.71	9.8	20
Vomiting (%)	0	5.71	2.08	1.82	0	20
Headache (%)	9.38	5.63	2.08	0	3.92	0
Dizziness (%)	0	3.61	1.03	1.45	1.75	0

SD, standard deviation.

There were 96 patients with previous psychiatric diagnoses (any one of PTSD, depression, anxiety, OCD, SUD, grief, attention deficit disorder (ADD) or attention deficit hyperactivity disorder (ADHD), eating disorder). In summary, these patients accounted for the majority of reported adverse events across all sessions (75% of sessions with nausea, 77.78% of sessions with vomiting, 83.33% of sessions with headache, and 76% of sessions with anxiousness).

### Elevated blood pressure

The average pre-session blood pressure was 128/84 (SD 15/11). About 36.85% of sessions had participants with elevated blood pressures prior to KaT, while 49.16% had elevated blood pressures post-KaT. The average post-session blood pressure was 130/87 (SD 19/15). Only nine participants required administration of clonidine for treatment of pre-KaT hypertension (mean dose 0.19 mg). Five participants had known hypertension controlled with pharmacotherapy. These participants accounted for 8.82% of 204 sessions that reported hypertension post-KaT.

Also, intramuscular and sublingual sessions did not largely differ in average pre-KaT blood pressure (128/84 and 126/84, respectively) and post-KaT blood pressure change from baseline (49.40% from 37.71% baseline; and 48.10% from 32.98% baseline, respectively). The average post-KaT blood pressure for the intramuscular and sublingual KaT groups were 130/86 (SD 19/14) and 132/88 (SD 20/17), respectively.

More males experienced post-KaT elevated blood pressure compared with female participants (Table 4).

As in Table 5, post-treatment elevated blood pressure was more prevalent among older participant cohorts, peaking in participants aged 61–70 years (71.15% of sessions) and likely descending in the 71- to 80-year-old cohort due to very small sample size (five sessions). Table 5 also indicates the post-session blood pressure values for each age group.

No other adverse events such as neurological symptoms, hallucinations, anaphylaxis, or respiratory depression were reported by participants or team members. No participants experienced adverse effects that precluded their participation in RTT-KaT.

### Impact of prior psychedelic experience

Altogether, there were 48 individuals who had any sort of previous psychedelic use; 21 of which with previous recreational or medical ketamine use. In summary, these patients accounted for a minority of reported adverse events across all sessions (22.73% of sessions with nausea, 0% of sessions with vomiting, 33.33% of sessions with headache, 12% of sessions with anxiousness, and 19.12% of sessions with post-KaT hypertension).

There were 35 patients with previous history of using other psychedelic drugs or practices that involve psychedelic use (any one of sweat lodge, psilocybin, lysergic acid diethylamide (LSD), 3,4-Methyl enedioxy methamphetamine (MDMA), 5-methoxy-N,N-dimethyltryptamine (5 MEO-DMT), ayahuasca, huachuma, or iboga). These patients also accounted for a minority of reported adverse events across all sessions (18.18% of sessions with nausea, 22.22% of sessions with vomiting, 16.67% of sessions with headache, and 24% of sessions with anxiousness.

## Discussion

This article highlights the safety and tolerability of sublingual and intramuscular ketamine in the RTT-KaT program as an alternative treatment modality for participants facing mental health issues. The majority of KaT participants only experienced transient adverse reactions, which were resolved through non-pharmacotherapy or pharmacotherapy.

### Trends in adverse events

The incidence of adverse events such as nausea, vomiting, headache, and dizziness were similar in our study compared with past studies of KaT using sublingual and intramuscular routes of administration.^7^ In particular, the rates of nausea and vomiting in this study were higher than in-home sublingual administration but lower than in-office sublingual or intramuscular routes of administration.^7^ Compared with previous studies describing the use of ketamine for procedural sedation, there were fewer rates of nausea and vomiting in this study.^23,24^

The use of medication prophylaxis has also proven to be effective in prevention of adverse events including nausea, vomiting, anxiousness, and hypertension, which is a helpful finding contributing to best practices in individual or group KaT in the future.

Headache as an adverse event for ketamine use has been documented elsewhere.^25^ In this study, one possible mechanism is that ketamine dosing resulted in rapid blood pressure elevation, which has been linked to headache manifestation.^26,27^ However, it is important to note that ketamine has also been an increasingly well-tolerated and effective emerging treatment for headaches.^28–31^ The *N*-methyl-d-aspartate (NMDA) receptors in the body are responsible for processing pain signals.^32^ When overstimulated, NMDA receptors can become excitotoxic causing headaches, such as migraine, and other pain disorders.^32^ Ketamine is an NMDA glutamate receptor antagonist and has been primarily hypothesized to decrease the intensity of neurotransmissions through the brain, thereby diminishing headaches.^32^

Certain demographic groups may have a higher tendency to be susceptible to adverse events such as nausea, vomiting, headache, and anxiousness. In particular, as seen in previous studies, the incidence of women experiencing nausea and vomiting whether it is postoperative or in terminal cancer patients has been shown to range between two-thirds and twofold that of men.^33–35^ Similar trends were also shown in this study. In addition, patients with psychiatric diagnoses such as depression and anxiety have been shown to have strong correlations with increased prevalence of nausea, vomiting, and headache in the community.^36,37^ This is again reflected in this study and has important implications for prophylactic medication and patient education among participants who enroll in KaT.

### Past psychedelic experience

KaT is a novel experience for many people given its relatively recent re-introduction into the healthcare space, as well as the limited number of clinics that offer the service. While ketamine tolerance with repeated use has been described in animal and human reports,^38–40^ new participants without any past psychedelic experience may feel somewhat apprehensive prior to their ketamine experience.^41^ This may lead to increased nausea and vomiting, which has been reported as related prior to other medical treatments.^42^

Thus, it is likely that familiarity with psychedelics promotes a more regulated experience, increasing safety and tolerability for participants. Although there is likely a benefit of past psychedelic experience in preventing adverse effects, our study population consisted of relatively few individuals with past psychedelic use (*n* = 48) compared with those without past use (*n* = 80). Thus, the directional but insignificant association found in this study between past psychedelic use and adverse effects may be a result of sample size variation among those with previous psychedelic use. We hypothesize that greater, more balanced sample sizes between these groups would provide sufficient power to reach significance.

It will be optimal for future KaT providers to consider the possible connection between decreased tolerability with being psychedelic-naive, especially for participants who are entirely new to psychedelic-assisted therapies. Pre-emptive therapeutic treatments should be provided such as a calm welcoming space, an option to listen to music, or use noise canceling headphones prior to receiving ketamine and talking to a counselor or nurse the day prior to KaT.^43,44^

### Elevated blood pressure

All patients were normotensive at baseline with the absence of hypertension prior to enrolling in the RTT-KaT program. The changes in blood pressure prior to KaT sessions could be due to anticipatory anxiety (also known as ‘white coat hypertension’).^45^ Ketamine is known to raise blood pressures as an expected side effect. The post-ketamine hypertension may be related to white coat hypertension, ketamine-related hypertension, or a combination of both. Our study’s documented increase of about 12% of elevated blood pressure from pre- to post-KaT is below that which is documented in literature.^46,47^

The systolic blood pressure cut-off of 150 mmHg accounted to some degree for white coat hypertension while still maintaining a safe upper limit for patients enrolled in the RTT-KaT program.^48,49^ The incidence of pre-treatment cut-offs for older age cohorts falling marginally near the upper limit was accounted for by vascular stiffness.^50^

The elevation of blood pressure related to therapeutic ketamine is transient and expected and need not be of concern in treating normotensive or well-controlled hypertension patients.^51–53^ The elevation in blood pressure is in a similar range as expected in exercise.^54,55^

### Strengths

This study reports on the reality of operating community-based KaT as opposed to controlled clinical trial environments. This type of setting provides valuable ecologically valid safety data for real-world repetition of clinical services.^56^

The way that RTT-KaT is structured, it likely promotes harm reduction by providing a safe alternative to those who might otherwise seek unregulated care. Furthermore, the CoP structure provides the safe relational space to facilitate and enhance integration, which is helpful to embody the insights that emerge during the psychedelic experience. Medical professionals and trained counselors are available to help prepare the participant both medically and psychosomatically for their psychedelic experiences. After each medicine session, integrated therapy allows participants to process their psychedelic experience and incorporate insights and realizations into life changes.

Documenting and publishing on community-based research allows for creation of similar programs and sets realistic expectations for existing group KaT programs. The majority of mental health work is done in the community by primary care providers and therapists and not in hospitals. Thus, this publication adds to the literature supporting ketamine-assisted therapy in the community.

## Limitations

Limitations of this article include reliance on medical or psychiatric diagnoses provided by the referring healthcare provider and the patient on their intake from prior to being enrolled in the RTT-KaT program. Although the results did not show a difference in rates of adverse effects between participants with and without a psychiatric diagnosis, previous studies indicate a link between psychiatric diagnoses and gastrointestinal or somatic symptoms.^37,57–59^ Our study population consisted of few individuals without past psychiatric diagnoses (*n* = 32) compared with those with past diagnoses (*n* = 96), with the former population being less than half of the latter. Thus, the insignificant association between past psychiatric diagnosis and adverse effects may be a result of sample size variation among those without previous psychiatric diagnosis. We hypothesize that greater, more balanced sample sizes between these groups would provide sufficient power to reach significance.

Some patients may not have had their diagnoses documented if they did not self-disclose and their referring health care provider did not disclose. Without considering the presenting diagnoses, the research team was not able to have a point of reference for the scoring of the validated psychiatric scales. This may have affected analyses on adverse effects post-treatment such as reported hypertension, nausea, and vomiting, which may be higher in patients with anxiety.^37,57,60^ Other somatic symptoms such as headache and pain may be higher in patients with depression.^58,59,61^ Other limitations include the limited time frame of follow-up and lack of generalizability beyond the specific groups involved. Underrepresented groups include marginalized populations such as Indigenous and other minority groups. The uneven number of participants by cohort and by age such that some results may have skewed analyses.

Another potential limitation of the study was our inability to assess long-term risks of KaT including potential for the development of nonmedical ketamine use. However, it is noteworthy that reviews of the literature on ketamine-assisted psychotherapies reported no evidence of transition from medicinal to illicit use.^5,62–66^ Nonetheless, future longitudinal studies and cohort designs will be required to address this issue more fully. In the meantime, the risk that recipients of ketamine therapy might develop problematic use patterns is speculative and not based on any evidence and as such should not be used as a rationale to deny treatment. Indeed, preliminary evidence suggests that KaT may counter problematic substance use.^5,62–65^

This study also does not investigate the long-term outcomes of RTT-KaT, as patients most often received an upper limit of three KaT sessions with intermittent alumni sessions thereafter. However, intravenous ketamine has been investigated extensively since the 1970s.^6,10,12^ According to one review on the long-term effects of ketamine, based on available data, the side effects of ketamine (e.g. nausea, dissociation, headache, and elevated hemodynamic metrics) are typically self-limited, transient, and mild.^67^ Intranasal ketamine has been associated with higher risk for lower urinary tract symptoms, including dysuria, yet severe bladder pathology has not been reported with typical ketamine-prescribing patterns for depression.^67^ Finally, ample data suggest that high-dosage ketamine use is associated with long-term cognitive impairment.^67^ However, recent clinical trials report stable or improving levels of cognition over time with appropriate ketamine usage.^67^

In addition, we acknowledge that certain outcomes had non-negligible proportions of missing data. A reason for this occurrence is due to the retrospective nature of the study, where there was no previous standardized protocol to prospectively collect outcomes of interest in this study. Furthermore, missing data in this study were not due to loss to follow-up (e.g. due to withdrawal from the program or mortality), given that zero participants had dropped out. Thus, the missing data in this study can be attributed to the practical expectation that there may be some degree of incomplete translation of clinical work in this practical program into retrievable chart data, given its retrospective design.

Finally, given the practical service-based nature of the RTT program, another limitation is that patients received ketamine via different routes of administration for reasons related to clinical judgment and patient preference, rather than to balance allocation of intramuscular *versus* sublingual ketamine administration. However, few studies to-date have investigated the safety of either route of ketamine administration in the novel setting of CoP group psychotherapy. This study preliminarily piloted whether safety and tolerability characteristics of sublingual or intramuscular ketamine generalize to this setting. Further clinical trials in this setting with more balanced study arms are needed to fully elucidate differences in safety and tolerability by administration route.

## Conclusion

While the absence of major adverse effects in this study among 448 sessions does not indicate the absolute lack of risk in other KaT sessions or programs, this study does provide information on excellent retention and general safety and acceptability of RTT-KaT. It also provides good indication of the occurrence of adverse effects and both non-pharmacological and pharmacological interventions, and the dosing required to treat common adverse effects such as nausea, headache, and hypertension during and post-KaT. The standardized model of RTT-KaT described above is the foundation for safe and effective practice.

## Supplemental Material

sj-docx-1-tpp-10.1177_20451253231171512 – Supplemental material for Safety and tolerability of intramuscular and sublingual ketamine for psychiatric treatment in the Roots To Thrive ketamine-assisted therapy program: a retrospective chart review Click here for additional data file. Supplemental material, sj-docx-1-tpp-10.1177_20451253231171512 for Safety and tolerability of intramuscular and sublingual ketamine for psychiatric treatment in the Roots To Thrive ketamine-assisted therapy program: a retrospective chart review by Vivian W.L. Tsang, Brendan Tao, Shannon Dames, Zach Walsh and Pam Kryskow in Therapeutic Advances in Psychopharmacology

## Declarations

**Ethics approval and consent to participate:** Cohorts 1–3 were exempted for quality improvement purposes through the Island Health Authority. Ethics approval was obtained from the Vancouver Island University Research Ethics Board (ethics approval number H20-30379) for the secondary use of Cohort 4 treatment data. Participant written consent for quality improvement and secondary use of data for research was obtained from all participants.

**Consent for publication:** Not applicable.

**Availability of data and materials:** Data and materials can be made available upon request to the lead author.
