# Supplementary material for: Safety and tolerability of intramuscular and sublingual ketamine for psychiatric treatment in the Roots To Thrive ketamine-assisted therapy program: a retrospective chart review
Source: Ther Adv Psychopharmacol. 2023 May 25;13:20451253231171512. doi: 10.1177/20451253231171512 (PMC10225955; doi:10.1177/20451253231171512)
Supplement: sj-docx-1-tpp-10.1177_20451253231171512 – Supplemental material for Safety and tolerability of intramuscular and sublingual ketamine for psychiatric treatment in the Roots To Thrive ketamine-assisted therapy program: a retrospective chart review [file sj-docx-1-tpp-10.1177_20451253231171512.docx]

**SUPPLEMENTARY MATERIALS**

As certain participants had more sessions than others, analyzing by individual reduces the weighting of one single over-represented participant on study estimates. However, this advantage is offset by a loss in study power and data resolution. Analysis by individual was conducted by first averaging the variable number of session entries per participant, before averaging across all individuals, thereby equalizing individual weightings for pooled estimates. The result of this calculation is a number between 0 and 1. In this scoring system (individual score [I-score]), 0 depicts that no individual experienced a given event in any of their sessions, while 1 depicts that all individuals experienced a given event in all their sessions.

**Supplementary Table S1: Overall baseline features stratified by gender (by individual)**

|  | **Male (N=30 individuals)** | **Female (N=98)** |
| --- | --- | --- |
| **Age (mean [SD])** | **49.21 (11.18)** | **46.04 (11.27)** |
| **Weight (mean [SD])** | **82.67 (11.33)** | **71.87 (15.76)** |
| **Pre-BP (mean [SD])** | **134.14/85.73 (SD:11.84/8.61)** | **126.27/83.43 (11.68/8.57)** |
| **Pre-Treatment HTN (I-score)** | **0.51** | **0.34** |

**Supplementary Table S2: Adverse effect measures stratified by gender (by individual)**

|  | **Male (n=30 individuals)** | **Female (n=98 individuals)** |
| --- | --- | --- |
| **Nausea (n [I-score])**  **Vomiting (n [I-score])**  **Headache (n [I-score])**  **Dizziness (n [%])** | **4 [0.075]**  **1 [0.11]**  **0**  **0 [0%]** | **29 [0.14]**  **7 [0.26]**  **10 [0.034]**  **5 [5.1%]** |
| **Post-elevated blood pressure (I-score)** | **0.64 (vs 0.51 baseline)** | **0.47 (vs 0.34 baseline)** |
| **Post-session blood pressure (mean [SD])** | **137/90 (SD 15/13)** | **129/86 (SD 13/19)** |

**RESULTS (BY INDIVIDUAL)**

**Summary of Adverse Effects**

Supplementary Table S1 depicts the baseline features of the 128 included participants. Supplementary Table S2 illustrates the occurrence of adverse effects, stratified by gender. Overall, the weighted I-scores for each adverse effect did not largely deviate from their corresponding by-session data points (shown in Table 4).

No other adverse events such as neurological symptoms, hallucinations, anaphylaxis, or respiratory depression were reported by participants or team members. No participants experienced adverse effects that precluded their participation in RTT-KaT.

**Stratification by Baseline Differences**

For comparison of intramuscular versus sublingual administration, no “by individual” analysis was conducted as individuals received both IM and SL treatments between sessions, meaning this data cannot be aggregated.

Gender was not significantly associated with nausea adverse effects (X2 = 2.69, p-value = 0.10), unlike by-session analysis. However, this nearly significant finding is likely a result of data aggregation of the session data into individual data, which decreased the sample size and statistical power compared to by-session analysis.

There were 96 patients with previous psychiatric diagnoses (any one of PTSD, depression, anxiety, OCD, SUD, Grief, ADD/ADHD, eating disorder) while 32 individuals had no previous diagnosis. From the former group, 38 individuals experienced any adverse effect, while from the latter group, 13 participants experienced any adverse effect. Past psychiatric diagnosis was not significantly associated with the development of any adverse effects (nausea, vomiting, headache, anxiousness) (X2 = 0.011, p-value = 0.92).

**Elevated Blood Pressure**

There were 5 participants with a known diagnosis of hypertension, stabilized by pharmacotherapy. A moderate proportion of individuals had had elevated blood pressures prior to KaT session (I-score: 0.38), and this proportion elevated mildly after KaT (I-score: 0.51% by individuals). The average post-session blood pressure was 131/87 (SD 14/10). Only 9 participants required administration of clonidine for treatment of pre-KaT hypertension (mean dose 0.19mg). As in Supplementary Table S1, more males experienced post-KaT elevated blood pressure across their sessions than female participants.

The highest recorded post-KaT blood pressure was 187/127 and only 2 individuals had systolic blood pressures in the 180s. 39 participants had systolic blood pressures in the 140s, 31 participants had systolic blood pressures in the 150s.

**Impact of Prior Psychedelic Experience**

Altogether, 48 individuals had any sort of previous psychedelic use. There were 21 participants specifically with previous recreational or medical ketamine use. As well, we identified 35 patients with previous history of using other psychedelic drugs or practices that involve psychedelic use (any one of sweat lodge, psilocybin, LSD, MDMA, 5 MEO-DMT, ayahuasca, huachuma, or iboga).

To account for individuals who reported adverse effects in multiple sessions, we identified 80 individuals with no previous psychedelic use (33 having experienced any adverse effect) and 48 individuals with previous psychedelic use (18 having experienced any adverse effect). Past psychedelic use was not significantly associated with the development of any adverse effects (nausea, vomiting, headache, or anxiousness; X2 = 0.054, p-value = 0.82).
